# A Novel Defect Inspection System Using Convolutional Neural Network for MEMS Pressure Sensors

**DOI:** 10.3390/jimaging8100268

**Published:** 2022-09-30

**Authors:** Mingxing Deng, Quanyong Zhang, Kun Zhang, Hui Li, Yikai Zhang, Wan Cao

**Affiliations:** 1 School of Automobile and Traffic Engineering, Wuhan University of Science and Technology, Wuhan 430065, China; 2 The Institute of Technological Sciences, Wuhan University, Wuhan 430072, China; 3 School of Power and Mechanical Engineering, Wuhan University, Wuhan 430072, China; 4 Wuhan FineMEMS Inc., Wuhan 430075, China

**Keywords:** MEMS pressure sensor, defect detection, convolutional neural network

## Abstract

Defect inspection using imaging-processing techniques, which detects and classifies manufacturing defects, plays a significant role in the quality control of microelectromechanical systems (MEMS) sensors in the semiconductor industry. However, high-precision classification and location are still challenging because the defect images that can be obtained are small and the scale of the different defects on the picture of the defect is different. Therefore, a simple, flexible, and efficient convolutional neural network (CNN) called accurate-detection CNN (ADCNN) to inspect MEMS pressure-sensor-chip packaging is proposed in this paper. The ADCNN is based on the faster region-based CNN, which improved the performance of the network by adding random-data augmentation and defect classifiers. Specifically, the ADCNN achieved a mean average precision of 92.39% and the defect classifier achieved a mean accuracy of 97.2%.

## 1. Introduction

A microelectromechanical system (MEMS) is miniaturized, compact, inexpensive, easy to integrate, and compatible with the standard process [1]. It has been widely used in industrial productions [2]. The quick development of electronic portable, wearable, or implantable devices increases the need to develop very small-sized integrated MEMS sensors [3].

MEMS pressure sensors have prominent advantages in weight and power consumption due to their characteristics [4]. Basov [5] proposed a mathematical model of a high-sensitivity pressure sensor with a novel electrical circuit utilizing a piezosensitive transistor differential amplifier with a negative feedback loop. The circuit based on a vertical NPN and a horizontal differential transistor amplifier was used to analyze and optimize the pressure sensitivity and output stability of a PNP transistor. Yu et al. [6] presented a piezoresistive absolute micro-pressure sensor, which was of great benefits for altitude location. Based on the ANSYS simulation results, the surface stress and deflection were analyzed. The sensor equation was established. Xu et al. [7] systematically analyzed and discussed the influence of the bending-stiffness distribution of the diaphragm on the stress-concentration characteristics of the pressuresensor chip. In order to improve the application range of the pressure sensor, Basov [8] designed a pressure sensor suitable for an ultra-low pressure range and optimized its structure. Based on the theoretical model and experimental characteristics of an ultra-high-sensitivity MEMS pressuresensor chip, Basov [9] proposed a novel circuit. A pressure-sensitive differential amplifier was adopted in the circuit and had a negative feedback loop based on two bipolar junction transistors.

However, some nonelectrical faults may occur in MEMS. Defect detection plays an essential role in reliability evaluation [10]. The method of deep learning is superior to traditional machine learning and has been widely used in various fields [11]. Because of its wide application, various deep-learning neural networks have been promoted. Lecun et al. [12] proposed a suitable network architecture that used a gradient-learning algorithm to synthesize complex decision surfaces. The classification of high-dimensional patterns such as handwritten characters was realized, and various handwriting-recognition methods were compared. Convolutional neural networks show the ability to surpass all other technologies. Simonyan et al. [13] proposed a classic visual-geometry group network to promote the development of deep learning. The performance of neural networks is improved by adding neural-network layers, but the cost is a serious shortcoming. More specifically, a deeper network means more parameters, which can easily cause overfitting, high computational expenses, and long training cycles. Parameters are related to the fully connected layer. Therefore, replacing the fully connected layer with sparse connections is the key to solving these problems [14]. The occurrence of the residual structure increases the number of network layers and improves the accuracy without the phenomenon of gradient dispersion and explosion. Some classical neural networks have been proposed. Krizhevsky et al. [15] first proposed the AlexNet neural network using the ReLu function as the activation function of neurons. By visualizing the middle layer result of AlexNet, Zeiler et al. [16] improved the model by reducing the convolution kernel. Szegedy et al. [17] combined the initial structure with the residual connection to improve the overall effect of the network. Huang et al. [18] proposed a dense convolutional neural network (CNN) that connects each layer with all previous layers. Focusing on the channel dimension of convolutional neural networks, Hu et al. [19] proposed a novel architectural unit, termed the "Squeeze-and-Excitation" (SE) block, by explicitly modeling the interdependencies between channel relationships to adaptively recalibrate the channel-wise eigenresponse. Howard et al. [20] proposed the mobileNet network, which is an efficient, lightweight, and streamlined network. The separable convolution was used in the depth direction of the network, and two hyperparameters, width multiplier and resolution multiplier, were used to balance the model speed and precision. Zhang et al. [21] proposed the ShuffleNet network, which was a computationally efficient CNN architecture specially designed for mobile devices with very limited computing power. The new architecture utilized two new operations, pointwise group convolution and channel shuffling, which greatly reduced computational cost while maintaining accuracy. Xie et al. [22] proposed a simple, highly modular network structure for image classification. The network was constructed by repeating a building block that aggregates a set of transitions with the same topology. Sandler et al. [23] proposed MobileNetV2 by removing the nonlinear activation layer behind the small-dimensional output layer. The inverted residual structure was used to ensure the expressiveness of the model and alleviate the degradation of features. The accuracy rate of the MobileNetV2 was 78% but the reduction of the numbers of network parameters reduced the difficulty of the deployment. Based on the traditional convolution-module design, Mehta et al. [24] proposed an efficient spatial-pyramid convolution module (ESP Module) that could reduce model computation, memory, and power consumption. The applicability of terminal devices was improved and the accuracy rate of the ESP Module was 85%. On the premise of reducing the number of parameters, the detection effect of the network was maintained. Based on Shufflenet V1, Ma et al. [25] proposed Shufflenet V2 by reducing the degree of network fragmentation. The number of groupings and element-by-element operations improves the speed and accuracy of the network. Mehta [26] proposed an EspNetv2 that replaced the original EspNet pointwise convolutions with group pointwise convolutions. The accuracy rate of the network was 89%. This network had good robustness and few parameters, which had a significant impact on the application.

Currently, the mainstream target-detection networks include the Faster Region-based Convolutional Neural Network (Faster RCNN) and You-Only-Look-Once (YOLO). The main difference between the two networks was that YOLO had a faster detection speed, whereas Faster RCNN had more accurate detection results [27,28]. He et al. [29] classified the defects of the steel surface with an accuracy of 99.67%; the MAP of defect inspection was 82.3%. However, the structure of the network was complex and unstable, and the MAP was still far from the industrial requirements. Some deep-learning algorithms were proposed for industrial inspections, but could not be fully adapted to the universal defects inspection. Therefore, in order to obtain better detection, a defect detection algorithm with low time cost needs to be developed to meet the defect characteristics of different products. Aiming at the defects in the manufacturing processes of the MEMS pressure sensor, accurate detection CNN (ADCNN) based on Faster RCNN is proposed in this paper.

First, the devices for packaging the MEMS pressure sensor and the packaging process of the pressure sensor are introduced. Chip damage, chip scratches, glue surface, gold-wire bonding, and aluminum-wire bonding are the defects that likely occur in the chip-packaging processes, which may cause failure of the performance of the MEMS pressure sensor. The types of defects for MEMS pressure sensors are provided by the MEMS pressure-sensor manufacturer. Second, an ADCNN network is proposed for MEMS pressure-sensor defect detection. The ADCNN is based on the Faster RCNN network, which improves the accuracy by using random-data augmentation and defect classifiers. Then, the training method and experimental results of the model are presented. Finally, the ADCNN is used to detect the defects of the MEMS pressure sensor.

## 2. System Overview

**Figure 1:**
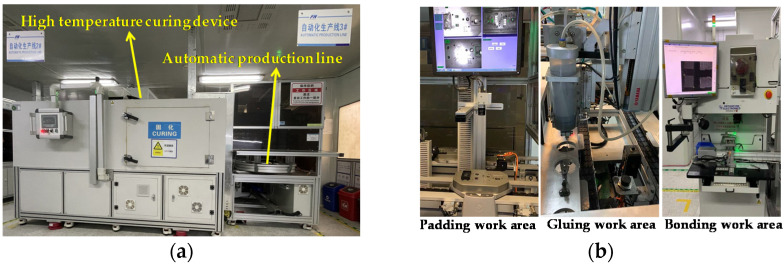
MEMS pressure-sensor packaging equipment. (**a**) High-temperature curing device and automatic production line. (**b**) Mechanical devices of automatic-production-line work area.

**Figure 2:**
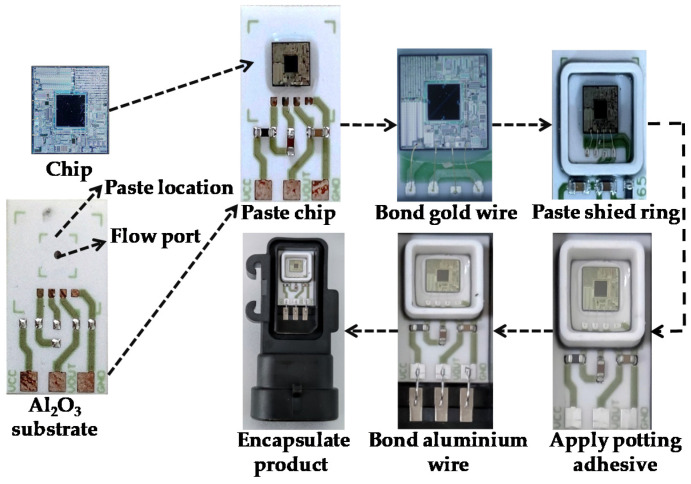
Packaging process of the MEMS pressure sensor.

Figure 1a shows the MEMS pressure-sensor packaging devices. A high-temperature curing device is been widely used for curing the glue used in the production process. An automatic production line is used for chip packaging. Figure 1b shows the mechanical device of the automatic production line. The device mainly includes three workstations. The padding workstation is used to pad raw materials. The gluing workstation is used to apply the glue. The bonding workstation is used to bond gold and aluminum wires. The MEMS pressure-sensor-chip packaging achieves automation by streamlining these workstations. Figure 2 shows the MEMS pressure-sensor packaging process. It consists of three main steps. First, a machine applies glue to the Al_2_O_3_ substrate and pastes the MEMS pressure-sensor chip. The process requires that the chip not have any damage or scratches. Second, the bonding workstation bonds the gold wire to connect the chip to the circuit on the Al_2_O_3_ substrate. Gold-wire bonding breaks may occur in the process. The next step is to reapply the glue and paste the shied adhesive. Then, the gluing workstation pours potting adhesive into the shied ring and bonds the aluminum wire. Glue-surface wrinkles and aluminum-wire bonding breaks may occur, resulting in MEMS pressure-sensor failure. Finally, the finished products are encapsulated.

**Figure 3:**
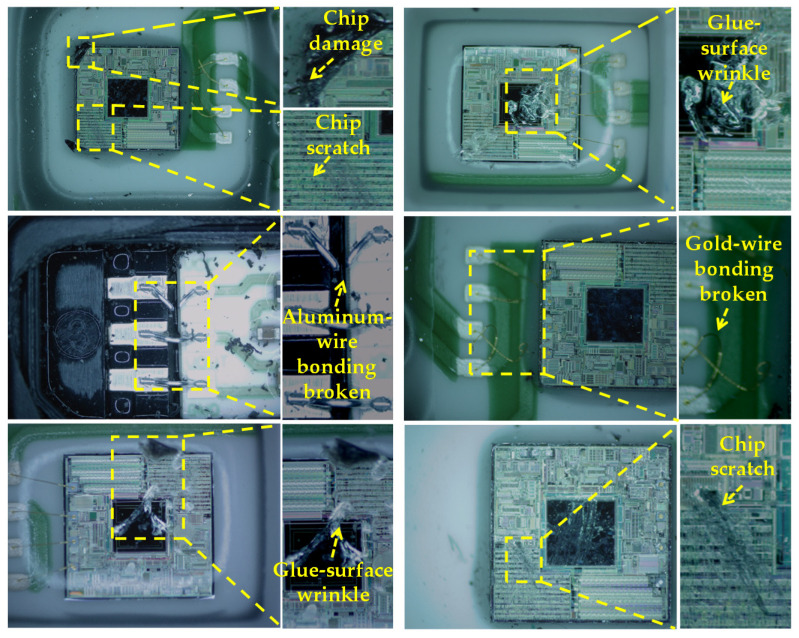
Five kinds of defect images of the MEMS pressure sensor from direct observation.

Figure 3 shows the sample defects of the MEMS pressure sensor. The image datasets, each with a resolution of 1000 × 800, were provided by Wuhan FineMEMS Inc. It contains five kinds of defects: chip scratch, chip damage, glue-surface wrinkle, broken gold-wire bonding, and broken aluminum-wire bonding. A chip scratch or chip damage could damage the internal circuits of the MEMS pressure-sensor chip. A glue-surface wrinkle could affect the air tightness of the MEMS pressure sensor. Broken gold-wire bonding and broken aluminum-wire bonding could lead to an open circuit, which could cause the performance failure of the MEMS sensor.

## 3. Proposed CNN

Defect detection is very important in industrial engineering. The purpose of this paper aims to determine all the defective objects in the image, and classify and locate them.

### 3.1. Improved Network Framework

The ADCNN is based on traditional Faster RCNN target detection. It improves the network to inspect the MEMS pressure-sensor chip-packaging process by changing the traditional convolution connection and adding defect classifiers and a random-data-augmentation module.

Traditional fast RCNN uses CNN to extract features and preset anchors points to obtain feature-layer and proposed anchor points. Then, the network obtains the location and classification. Traditional Faster RCNN extracts features and uses feature layers and suggested anchors to preset the anchor in the detection anchor to obtain the feature layer and suggested anchor of the target. However, the defects in MEMS pressure-sensor chips are different in size and scale. The number of defect images is limited. Traditional Faster RCNN cannot detect these different scale defects in a single image.

**Figure 4:**
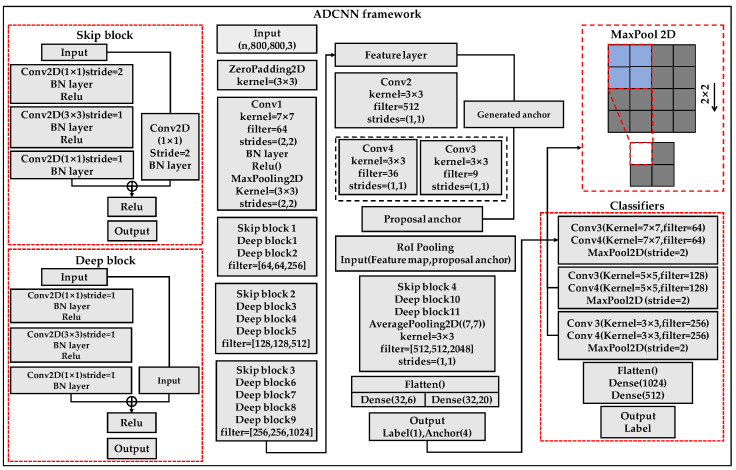
Framework of the ADCNN.

Figure 4 shows the detail of the ADCNN. The ADCNN comprises a detection location and classifier parts. The preliminary improvement changes the traditional convolution connection by two structures called skip and deep blocks in the detection part. The function of the skip block is to change the size of features extracted from defective images. It contains two repeated 1 × 1 convolutions, where strides are doubled. One of the convolutions is followed by a 3 × 3 convolution and 1 × 1 convolution, where strides are single. Each convolution is followed by batch-normalization layers and ReLu activation functions to prevent gradient explosion and vanishing. Features are put into two 1 × 1 convolutions, and then the convolved features are added. The deep block increases the number of network layers. It contains two repeated 1 × 1 convolutions and a 3 × 3 convolution. Strides of convolutions are single in the deep block. Extracted features and inputs are directly added. Two structures make the network better extract the details of defect images.

The second improvement is that the ADCNN adds defect classifiers to the traditional Faster RCNN. The improved traditional network is used to first detect the possible locations of smaller-scale defects. According to these locked positions, the defect classifier identifies further defects. The classifier part contains two repeated 7 × 7 convolutions, 5 × 5 convolutions, and 3 × 3 convolutions. Each repeated convolution is followed by max-pool layers. Classifiers can more effectively extract the features of detection targets by using convolutions of different sizes. The defect classifiers can improve the accuracy of the whole network detection.

### 3.2. Workflow of the Network

**Figure 5:**
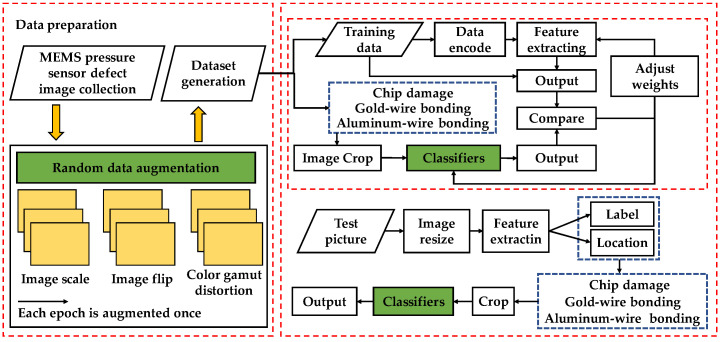
Workflow of the ADCNN for dealing with the defect images.

Figure 5 shows the workflow of the ADCNN. The implementation of target detection depends on a special detection dataset containing expensive manual annotations. Defect images are obtained from the MEMS pressure-sensor manufacturer and labeled. A total of 6707 defect images are taken from the production line of Wuhan FineMEMS Inc. The labeled data are augmented in each training epoch. The defect images are input to the ADCNN, and these images are processed to adapt to the input form of the network. This process is called data encoding.

The detection-location part of the ADCNN obtains defect images and outputs location and classification. The classifier part of the ADCNN obtains the output in the detection-location part. The network deals with different kinds of defects in different ways. For large-scale defects such as glue-surface wrinkling and chip scratching, the detected locations and classifications are directly output by the network. For gold-wire-bonding and aluminum-wire-bonding defects, whether the bonding effect is qualified needs to be determined by classifiers. Small-scale defects for chip damage need to be further identified by the classifier. By detecting the position information of the positioning part as the special input of the classifier, the defect images of gold-wire bonding, aluminum-wire bonding, and chip damage are processed by the network to obtain the classification results. The results of the classification and location in the detection are output.

## 4. Training and Validation

### 4.1. Data Augmentation

**Figure 6:**
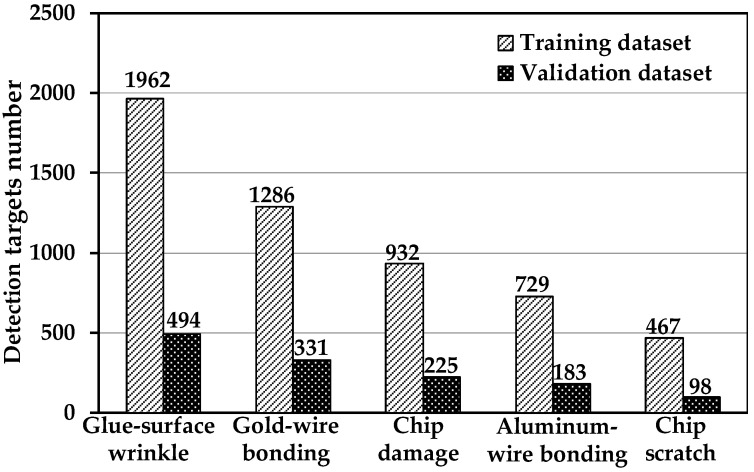
Numbers of five detection targets of MEMS pressure sensors.

**Figure 7:**
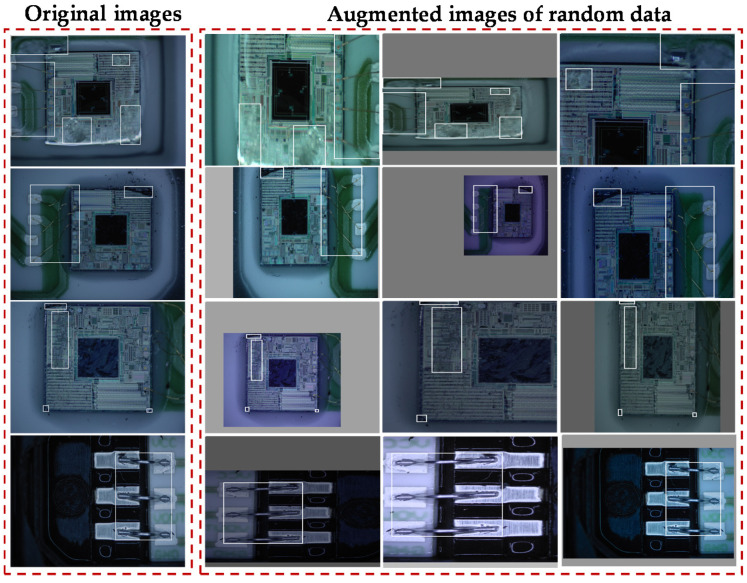
Data augmentation of five kinds of defect images of the MEMS pressure sensor in Figure 3.

Figure 6 shows the distribution of the defect datasets. The distribution is uneven and the number of defect images is limited. Random-data augmentation is applied to solve these problems. Random-data augmentation includes image scale, flip, and color-gamut distortion. The data-augmentation parameters are random in each training epoch. Thus, it significantly enhances data diversity and effectively avoids overfitting. Figure 7 shows the results from random-data augmentation. The size-scaling range of the defect image was between 0.9 and 0.5 of the original size. The flip angle was between 0 and 360 degrees. Gamut distortion was used to generate a new image by randomly adjusting the saturation, brightness, and contrast of the original images. The original image size, color gamut, and background were changed to obtain more defect images and to improve network robustness.

### 4.2. Training Method

The training process of the ADCNN is divided into three parts. First, the network is trained to obtain the proposal anchors. Then, the network is trained to obtain the detection location and the first classification result using the proposal anchors. Finally, the classifiers are trained to obtain the detection results.

The network is optimized by the gradient descent with the loss function as the objective function. The loss function includes classification loss and regression loss. The *L_cls_*_1_ classification loss is used to train parameters, which determines whether the preset anchors contain targets on MEMS pressure sensors. Conversely, the *L_reg_* regression loss is used to train parameters, which adjusts the location of the preset anchor on the MEMS pressure sensor. It is described as follows.

(1) $$L\left(p_{i},t_{i}\right)=\frac{1}{N_{cls}}L_{cls1}+\lambda \frac{1}{N_{reg}}\sum_{i}p_{i}^{*}L_{reg}\left(t_{i},t_{i}^{*}\right)$$

(2) $$L_{cls1}\left(p_{i},p_{i}^{*}\right)=\underset{i}{\sum }-log\left[p_{i}^{*}p_{i}+\left(1-p_{i}^{*}\right)\left(1-p_{i}\right)\right]$$

(3) $$L_{reg}=\left\{\begin{array}{c} 0.5x^{2},\;\;\left|x\right|<1\\ \left|x\right|-0.5,\;\;x\geq 1\;or\;x\leq -1 \end{array}\right.$$

where *i* is the anchor index of the batch and *p_i_* is the predicted probability of the target classification. $t_{i}$ is the predicted coordinate-adjustment value and $t_{i}^{*}$ is the true coordinate-adjustment value. *λ* is the weight, which balances classification loss and regression loss, and *x* is the value of the difference between $t_{i}$ and $t_{i}^{*}$. $p_{i}^{*}$ is a binary indicator, indicating whether the anchor contains the real detection target, and *N_cls_* is the number of classification-training batches. *N_reg_* is the number of regression-training batches. The adjustment parameters of the preset anchors are obtained by training networks, and the proposal anchors are obtained by adjusting parameters. Using the coincidence degree between the proposed and true anchors eliminates the proposal anchors with a low coincidence degree.

Then, the network is trained to obtain the coordinate-adjustment information of the proposal anchors and the classification results of detection targets in the detection-location part. The loss function is the same as in the previous loss function except for *L_cls_*_2_. The *L_cls_*_2_ loss is used to train parameters, which determines the classification results of the target on MEMS pressure sensors. It is described as follows.

(4) $$L\left(y_{ij},t_{i},p_{ij},p_{i}\right)=L_{cls2}+\lambda \frac{1}{N_{reg}}\sum_{i}p_{i}^{*}L_{reg}\left(t_{i},t_{i}^{*}\right)$$

(5) $$L_{cls2}\left(y_{ij},p_{ij}\right)=-\frac{1}{N_{cls}}\overset{N_{cls1}}{\underset{i=1}{\sum }}\overset{M}{\underset{j=1}{\sum }}y_{ij}\log\left(p_{ij}\right)$$

where *M* is the number of possible classifications. *y_ij_* is a binary indicator, indicating whether the classification *j* is the real classification of input instance *x_i_*. *p_ij_* is the probability that the network predicts that the input instance *x_i_* belongs to classification *j*.

The final step is to train classifiers for gold-wire bonding, aluminum-wire bonding, and chip damage. Classifier training is the same as the above training, and the loss function is the *L_cls_*_2_ function.

### 4.3. Training Results

**Figure 8:**
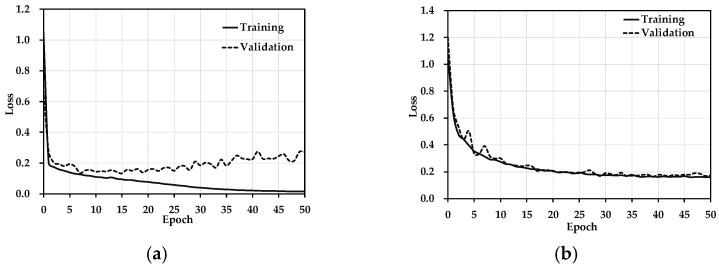
The MEMS pressure sensor detects the total loss of the positioning network. (**a**) Loss before data augmentation. (**b**) Loss after data augmentation.

Figure 8a shows the loss before data augmentation. There was over-fitting in training. Because the data set was very small, the network could not achieving good training results. Figure 8b shows the loss after data augmentation. The problem of overfitting was solved.

## 5. MEMS Defect Detection

**Figure 9:**
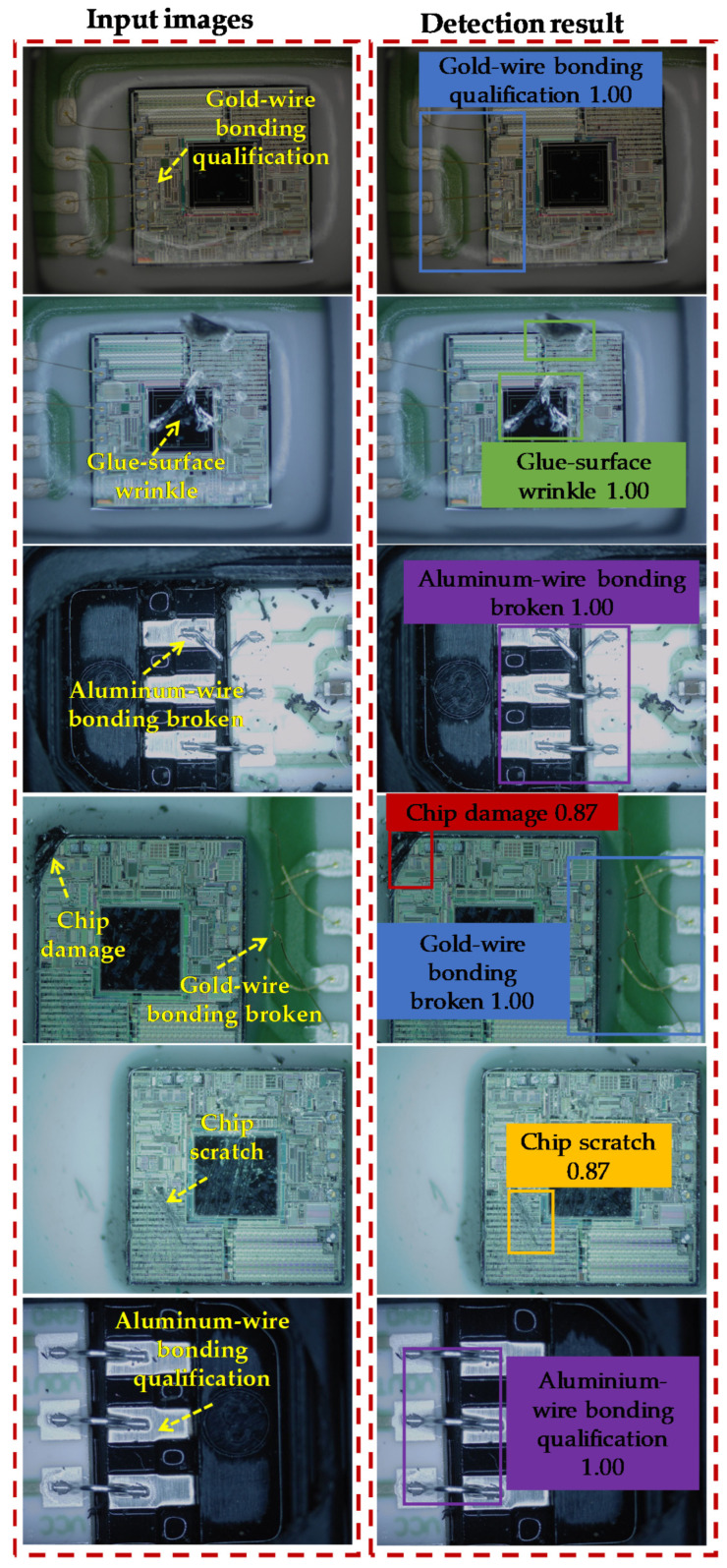
Defect-detection results of the MEMS pressure sensor based on the ADCNN.

Figure 9 shows the defect-detection results of the MEMS pressure sensor. The numbers are the probability of the ADCNN detecting the different defects. The ADCNN could detect gold-wire bonding, glue-surface wrinkles, aluminum-wire bonding, chip damage, and chip scratches in the packaging process of the MEMS pressure-sensor chip.

Average accuracy (*AP*) is used to evaluate the results of testing experiments. *AP* is a good trade-off between accuracy and recall, which are two important testing indexes. These indexes are defined as follows.

(6) $$Precision=\frac{TP}{TP+FP}$$

(7) $$Recall=\frac{TP}{TP+FN}$$

(8) $$AP=\frac{Precision+Recall}{2}$$

where *TP*, *FP*, and *FN* represent the number of true positives, false positives, and false negatives, respectively. The results of the defect detection were obtained for the MEMS pressure-sensor dataset. They include the mean average precision (MAP) and the accuracy in the detection location and classifier. Table 1 shows the target-detection statistics before network optimization. The MAP only achieved 89.6%, and the *AP* of chip damage only achieved 65.8%. The *AP*s of chip scratches, gold-wire bonding, and aluminum-wire bonding were high.

**Figure 10:**
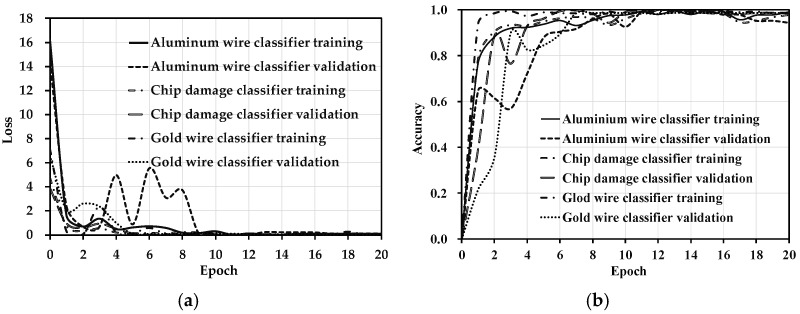
MEMS pressure-sensor detection-classification network. (**a**) Loss. (**b**) Accuracy.

In order to obtain high performance of defect identification and classification, the Adam optimizer was used with a learning rate of 0.05 and a batch number of 2. These parameters were determined based on the characteristics of the dataset. Table 2 shows the target-detection statistics after network optimization. The MAP achieved 92.4%. The random-data augmentation greatly alleviated the problem of fewer defects in the MEMS pressure sensor and the MAP of network was improved by optimization of the network. Recall of chip damage was 80%, but the precision was only 75.8%. Other defects scattered the attention of the network, which may have caused low precision of chip-damage detection. The network trained classifiers to solve this problem. Figure 10a shows the loss of classifiers. As shown in Figure 10a, the performances were excellent because overfitting did not occur in training. Figure 10b shows the accuracy of classifiers, where the average accuracy of classifiers was 97.2%. Since there is currently no research that combines deep learning and MEMS pressure-sensor chip-defect detection, it was compared with the research on steel-surface detection using deep learning. He et al. [29] classified the defects of a steel surface with an accuracy of 99.67%, and the MAP of defect inspection was 82.3%. Therefore, the low accuracy of chip damage was effectively solved by the classifiers. In addition, the stability of the network was improved by locating the aluminum-wire-bonding area and the gold-wire-bonding area, and checking the defects by classifiers.

It can be seen from Table 3 that the average detection accuracy of the network used in this work was 92.4%, and compared with the Faster RCNN, YOLOv3, YOLOv4 networks, the average detection-accuracy values were improved by 2.6%, 9.4%, and 1.1%, respectively. The detection time of a single defect picture was 68 ms, which meets the requirements of defect detection for a production line. Compared with other networks, the training results of the ADCNN were more accurate, and the detection time of a single image met the test requirements.

## 6. Conclusions

In this paper, a defect-detection system for MEMS pressure-sensor chip packaging is proposed. The system can obtain the specific category and detailed location of the defect. The ADCNN improves on the Faster RCNN framework with a skip block and a deep block. Additionally, it adds a defect classifier and data augmentation to improve the accuracy of the network. The experiments of the ADCNN on the MEMS pressure-sensor defect dataset show that the network achieved 92.4% MAP for the defect detection. These results demonstrate a high level in the industry. Future studies will focus on the following two directions. Firstly, because of the expensive manuals and symbols in the detection data set, a generative countermeasure network should be developed. Secondly, CNN is used for feedback control of the MEMS pressure-sensor chip package so as to obtain a more intelligent system.

## Figures and Tables

**Table 1:** Statistics of target-detection results without network optimization.

Detection Classes	*Recall* (%)	*Precision* (%)	*AP* (%)	MAP (%)
Chip scratch	100.0	95.2	98.2	89.6
Chip damage	94.5	60.9	65.8	
Gold-wire bonding	99.7	98.2	99.7	
Glue-surface wrinkles	82.0	98.8	84.3	
Aluminum-wire bonding	100.0	100.0	100.0	

**Table 2:** Statistics of target-detection results with network optimization.

Detection Classes	*Recall* (%)	*Precision* (%)	*AP* (%)	MAP (%)
Chip scratch	96.1	86.8	92.3	92.4
Chip damage	80.0	75.8	71.5	
Gold-wire bonding	98.3	82.7	98.8	
Glue-surface wrinkles	88.1	93.5	95.4	
Aluminum-wire bonding	100.0	100.0	100.0	

**Table 3:** Comparison of detection results of different networks.

Network	*AP*/%					MAP /%	Single-Picture Detection Time/ms
	Chip Scratch	Chip Damage	Gold-Wire Bonding	Glue-Surface Wrinkles	Aluminum-Wire Bonding		
Faster RCNN	90.5	89.6	93.5	85.7	89.9	89.8	51
YOLOv3	85.4	79.9	83.0	87.6	81.8	83.0	45
YOLOv4	92.8	82.7	95.5	93.2	92.4	91.3	42
ADCNN (This work)	92.3	91.2	98.8	95.4	98.4	92.4	68

## Data Availability

Not applicable.
